# Role of *Fusobacteria* in the serrated pathway of colorectal carcinogenesis

**DOI:** 10.1038/srep25271

**Published:** 2016-04-29

**Authors:** Chan Hyuk Park, Dong Soo Han, Young-Ha Oh, A-reum Lee, Yu-ra Lee, Chang Soo Eun

**Affiliations:** 1 Department of Internal Medicine, Hanyang University Guri Hospital, Hanyang University College of Medicine, Guri 11923, Korea; 2 Department of Pathology, Hanyang University Guri Hospital, Hanyang University College of Medicine, Guri 11923, Korea

## Abstract

*Fusobacteria* are associated with colorectal cancer (CRC) and are amplified during colorectal carcinogenesis. Compared to the adenoma-carcinoma sequence of carcinogenesis, serrated neoplasm has distinct clinical features and a different molecular background. We aimed to compare the gut microbiome between tubular adenoma (TA) and sessile serrated adenoma/polyp (SSA/P). Patients with TA, SSA/P, or CRC were recruited. Three pieces of colorectal mucosal tissue were obtained from each patient by endoscopic biopsy. 16S rRNA gene pyrosequencing and phylogenetic investigation of communities by reconstruction of unobserved states (PICRUSt) were performed. Among 26 enrolled patients, 8, 10, and 8 had TA, SSA/P, and CRC, respectively. The relative abundance of *Fusobacteria* did not differ significantly between the TA and SSA/P groups (4.3% and 1.9%, *P* = 0.739) but was higher in the CRC group (33.8%) than in the TA or SSA/P group, respectively (TA vs. CRC, *P* = 0.002, false discovery rate [FDR] = 0.023; SSA/P vs. CRC, *P* < 0.001, FDR = 0.001). PICRUSt revealed that most functions in the TA metagenome were similar to those in the SSA/P metagenome. The gut microbiome, including relative abundance of *Fusobacteria*, did not differ between TA and SSA/P, suggesting that *Fusobacteria* may contribute to both the serrated pathway and the adenoma-carcinoma sequence.

Most colorectal cancers (CRCs) are preceded by dysplastic adenomas that can progress to malignancy, a process known as the adenoma-carcinoma sequence^1^. Accumulation of genetic changes, including loss of the tumor suppressor gene *adenomatous polyposis coli (APC)* followed by activating and inactivating mutations in *KRAS*, *PIK3CA*, and *TP53*^2^, is usually required for the development of CRC. Additionally, it has been suggested that an altered gut microbiome may affect the development of CRC through interaction with the innate immune system and other host factors^3^,^4^,^5^. *Fusobacterium* species are in the spotlight in the development of CRC because many studies have shown that *Fusobacterium* is abundant in CRC tissue compared to adjacent normal tissue using next-generation sequencing^6^,^7^,^8^. In addition, Kostic *et al.* demonstrated that the *F. nucleatum* induced development of colonic tumor in *APC*^*Min/*+^mice. These results imply that *Fusobacteria* can affect the early stage of the adenoma-carcinoma sequence, such as *APC* mutated adenoma.

In addition to TA, serrated lesions are precursors of CRCs that exhibit hypermethylation and may account for one-third of all CRCs^9^. Sessile serrated adenoma/polyp (SSA/P) has distinct endoscopic features including a mucus cap, a color that is usually similar to normal mucosa, and indistinct edges^9^. Colorectal carcinogenesis of SSA/P is regarded as the results of epigenetic alterations in genes involving cellular proliferation and differentiation, caused by hypermethylation of promoter DNA, in addition to genetic mutations including *BRAF*^9^. The gut microbiome may also contribute to the serrated polyp-carcinoma sequence as well as the adenoma-carcinoma sequence because DNA methylation is closely related to the gut microbiome^10^,^11^. Nevertheless, the role of *Fusobacteria* in the serrated polyp-carcinoma sequence has not been fully evaluated. To identify whether microbial composition, including *Fusobacteria*, is different between tubular adenoma (TA) and SSA/P, we planned to compare the gut microbiome between those two types of CRC precursor. If *Fusobacteria* is also associated with the serrated polyp-carcinoma sequence, the microbial composition of SSA/P, including relative abundance of *Fusobacteria*, may be similar to that of TA. In this study we compared the gut microbiome among three different diseases—TA, SSA/P, and CRC—using 16S rRNA gene pyrosequencing.

## Results

### Baseline characteristics and microbiome reads

Table 1 shows baseline patient characteristics and microbiome reads. Among 26 enrolled patients, 8 had CRC, 8 had TA, and 10 had SSA/P. Median age was 68, 54, and 63 years in the TA, SSA/P, and CRC group, respectively (*P* = 0.119). The proportion of males did not differ significantly among the groups (*P* = 0.244). Five (62.5%) TAs and 7 (70.0%) SSA/Ps were in the proximal colon, whereas all lesions in the CRC group were in the distal colon.

A total of 136,414 read were obtained from 26 enrolled patients through 16S rRNA gene pyrosequencing. The median number of reads was 5,540, 6,162 and 4,969 in TA, SSA/P, and CRC groups, respectively (*P* = 0.247). Median operational taxonomic units (OTUs), Chao1 estimator, and Shannon’s and Simpson’s diversity index did not differ among the groups.

### Relative bacterial abundance

**Figure 1:**
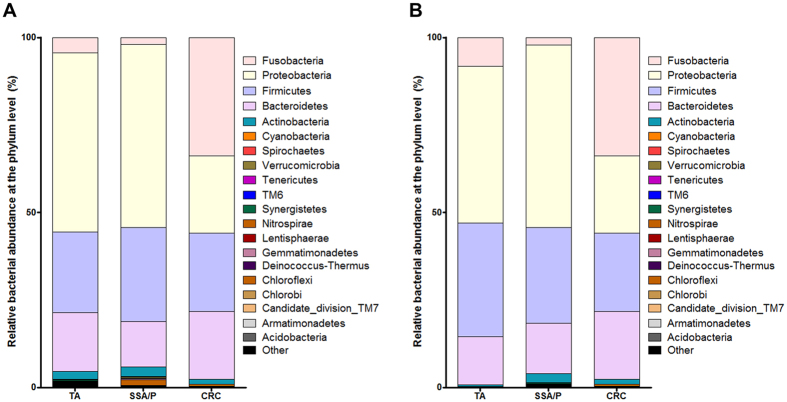
Relative bacterial abundance at the phylum level for all lesions (**A**) and only lesions located in the distal colon (**B**).

Figure 1A shows relative bacterial abundance at the phylum level for each group. The five most abundant phyla were *Fusobacteria*, *Firmicutes*, *Proteobacteria*, *Bacteroidetes*, and *Actinobacteria* in all three groups. Relative abundance of *Fusobacteria* did not differ between the TA and SSA/P group (4.3% and 1.9%, *P* = 0.739), but was significantly higher in the CRC group (33.8%) than in the TA or SSA/P group, respectively (Table 2; TA vs. CRC, *P* = 0.002, FDR = 0.023; SSA/P vs. CRC, *P* < 0.001, FDR = 0.001).

Because all lesions of the CRC group were in the distal colon, we performed a subgroup analysis for lesions located in the distal colon. As shown in Fig. 1B, relative abundance of *Fusobacteria* in the CRC group was higher than that in the TA or SSA/P group. Bacterial abundance at the phylum level was not associated with tumor location.

### Relative abundance of *Fusobacteria* in each tissue

**Figure 2:**
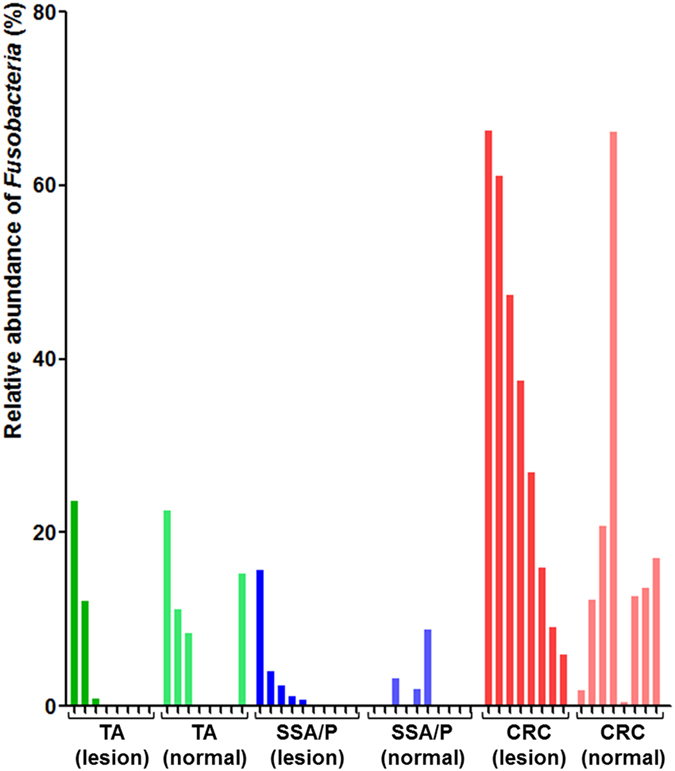
Relative abundance of *Fusobacteria* in each sample. Samples were grouped by disease and biopsy site (lesion vs. adjacent normal tissue). TA, tubular adenoma; SSA/P, sessile serrated adenoma/polyp; CRC, colorectal cancer.

Figure 2 shows relative abundance of *Fusobacteria* in each tissue. Although the relative abundance of *Fusobacteria* varied, 37.5% (3 of 8) and 50.0% (5 of 10) of lesions contained *Fusobacteria* in the TA and SSA/P group, respectively. In the CRC group, *Fusobacteria* was identified in all lesions. Interestingly, *Fusobacteria* was identified not only in the lesions but also in adjacent normal tissues. Four (50.0%), three (30.0%), and eight (100.0%) adjacent normal tissues in the TA, SSA/P, and CRC group, respectively, showed the presence of *Fusobacteria*.

### Principal coordinates analysis

**Figure 3:**
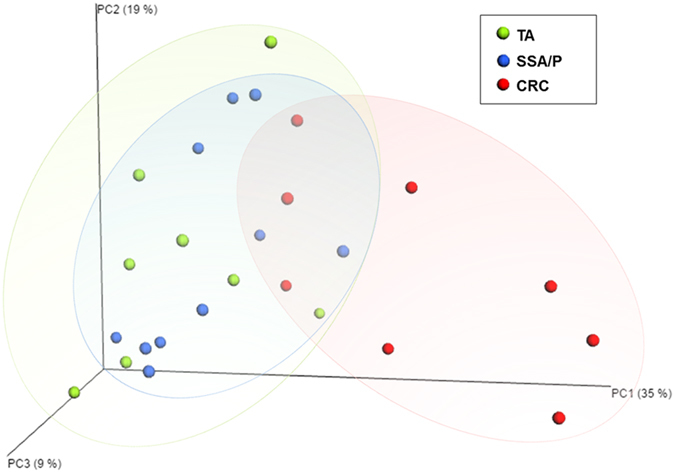
Principal coordinate analysis plot. The plots shows the clustering pattern among the TA (green), SSA/P (blue), and CRC (red) groups based on a principal coordinates analysis. TA, tubular adenoma; SSA/P, sessile serrated adenoma/polyp; CRC, colorectal cancer.

PCoA was performed to cluster the communities along axes of maximal variance (Fig. 3). The maximum variations were 35.4% and 18.7% in PCo1 and PCo2, respectively. No difference was observed between TAs and SSA/Ps. Bacterial communities in the TA or SSA/P group showed distinct patterns from those in the CRC group.

### Functional composition of the microbiome

After prediction of the microbial metagenome from the results of 16S rRNA gene pyrosequencing using PICRUSt, weighted NSTI was calculated in order to assess the reliability of the predicted metagenome. The predicted metagenome in all three groups showed excellent weighted NSTI (median [IQR]; TA, 0.050 [0.037–0.080]; SSA/P, 0.063 [0.036–0.091]; CRC, 0.063 [0.047–0.079]; *P* = 0.828)^12^.

Relative abundance of predicted function in each metagenome was demonstrated according to the level 1 KEGG module (Table 3). Although FDR was not significant, the relative abundance of the cellular processes category was higher in the TA (3.3%) and SSA/P (3.0%) groups than in the CRC group (2.3%) (TA vs. CRC, *P* = 0.010, FDR = 0.088; SSA/P vs. CRC, *P* = 0.027, FDR = 0.208). In contrast, the relative abundance of the genetic information processing category tended to be higher in the CRC group (19.4%) than in the TA (17.7%) and SSA/P (17.8%) groups (TA vs. CRC, *P* = 0.038, FDR = 0.088; SSA/P vs. CRC, *P* = 0.122, FDR = 0.208).

**Figure 4:**
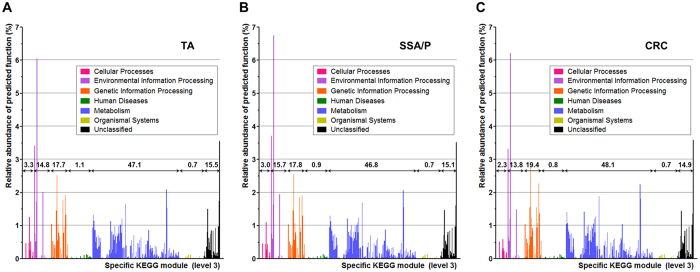
Relative abundance of functions predicted by phylogenetic investigation of communities by reconstruction of unobserved states. (**A**) TA, (**B**) SSA/P, and (**C**) CRC. Values represent percentage of relative abundance of each level 1 KEGG module. TA, tubular adenoma; SSA/P, sessile serrated adenoma/polyp; CRC, colorectal cancer; KEGG, Kyoto Encyclopedia of Genes and Genomes.

The relative abundance of functional category according to the level 3 KEGG module is illustrated in Fig. 4. Overall, distribution of each predictive function at the level 3 KEGG module did not differ among the three groups. However, several functions of the cellular process category including bacterial chemotaxis, bacterial motility proteins, and flagella assembly tended to be highly expressed in the TA and SSA/P metagenomes compared to the CRC metagenomes. On the contrary, several functions of the genetic information processing category including RNA degradation, DNA repair and recombination proteins, and mismatch repair tended to be more highly expressed in the CRC metagenome than in the TA and SSA/P metagenomes. Detailed relative abundance of predicted functions for specific level 3 KEGG modules is shown in supplementary Dataset 1.

## Discussion

Many studies have revealed that *Fusobacteria* are prevalent in CRCs compared to adjacent normal tissues^6^,^7^,^8^. It has been shown that *Fusobacteria* are present in colorectal premalignant lesions including TA and SSA/P although the relative abundance is lower than in CRC tissues^13^. Moreover, it was shown that abundance of *F. nucleatum* in CRC tissue was associated with a lower density of CD3+ T cell^14^. It has been suggested that Gut bacteria including *Fusobacteria* may have an influence on the development of CRC through interaction with the innate immune system or host factors^3^. These evidences support that *Fusobacteria* may have a distinct role in the carcinogenesis of the colorectum.

Recently, Kostic *et al.* demonstrated that *F. nucleatum* can induce intestinal tumorigenesis in *APC*^*Min/*+^mice, and showed that *Fusobacteria* can affect the early stage of the adenoma-carcinoma sequence such as *APC*-mutated adenoma. However, the influence of *Fusobacteria* on CRC development through the serrated pathway remained relatively unknown. Compared to the adenoma-carcinoma sequence, sessile serrated adenoma/polyp has an obviously different pathway to the development of CRC^1^,^9^,^15^,^16^. For example, abnormal hypermethylation of promoter CpG island is common in the serrated pathway to CRC^9^. In addition, *BRAF* mutations are strongly associated with CpG island methylator phenotype (CIMP)-high CRCs, acting as an alternative to the *KRAS* mutations that commonly occur in chromosomal instability cancers^9^. Moreover, *APC* mutation is relatively rare in SSA/Ps^15^. We hypothesized that if *Fusobacteria* are not associated with SSA/P, the gut microbiome, including relative abundance of *Fusobacteria*, might differ between TA and SSA/P tissues. In our study, however, the relative abundance of *Fusobacteria* did not differ between the TA and SSA/P groups. PCoA also showed that the gut microbiome in the TAs was more similar to that of the SSA/Ps than the CRCs. These results suggested that *Fusobacteria* is a possible contributor to both the adenoma-carcinoma sequence and the serrated polyp-carcinoma sequence.

In this study, the relative abundance of *Fusobacteria* in the TA or SSA/P groups was lower than in the CRC group. Considering that *Fusobacteria* are extremely rare in healthy individuals^17^, their abundance in 37.5–50% of lesions may be higher than the relative abundance of *Fusobacteria* in normal colorectal mucosal of healthy individuals. These findings were consistent with previous studies. One recent study by Ito *et al.* using quantitative polymerase chain reaction for *F. nucleatum* in formalin-fixed paraffin-embedded tumor tissues showed that only 30–40% of serrated adenomas expressed positivity for *F. nucleatum*^13^. Additionally, *F. nucleatum* expression did not differ between serrated and non-serrated adenomas. Moreover, *F. nucleatum* was more expressed in CRC tissues than in both serrated and non-serrated adenomas. Based on their results, Ito *et al.* concluded that expression of *F. nucleatum* may contribute to the progression of colorectal neoplasia.

Differences in the gut microbiome, including the relative abundance of *Fusobacteria*, between TA or SSA/P and CRC can be explained by the physiologic and metabolic changes that occur during colon carcinogenesis, including changes in colonic barrier function and rupture and bleeding of the colonic epithelium^18^. For example, *Bacteroides fragilis* (ETBF) strains produce *B. fragilis* metalloprotease toxin^19^,^20^. This metalloprotease facilitates cleavage of E-cadherin, that is tumor suppressor protein, in intestinal epitheliums, causing cell proliferation and permeabilization of the intestinal barrier^21^. As a result of changes in colonic barrier permeability and cellular metabolism, *Fusobacteria* might be more prevalent in CRC tissues than in adenomas^18^.

One interesting finding of our study is that *Fusobacteria* was identified in the adjacent normal tissues as well as colorectal neoplasms. The relative abundance of *Fusobacteria* between lesions and adjacent normal tissues was similar in each group. This finding can be explained by the field cancerization effect, i.e., a field of cellular and molecular alterations that predisposes to the development of neoplasms within that territory^22^,^23^. Other studies on gut microbiomes in patients with CRC also showed that *Fusobacteria* are abundant in adjacent normal tissues as well as CRC tissues^24^.

An additional merit of our study is functional analysis for the predicted metagenome using PICRUSt in TAs, SSA/Ps, and CRCs. Although statistical power was not achieved because of the small sample size, a tendency for functional differences among the three metagenomes could be identified. Overall, the relative abundance of the cellular processes category including bacterial chemotaxis, bacterial motility proteins, and flagella assembly tended to be higher in the TA and SSA/P groups, whereas that of the genetic information processing category including RNA degradation, DNA repair and recombination proteins, and mismatch repair tended to be higher in the CRC group. These results, however, were metagenomic inferences without statistical significance. Therefore, more experimental studies on these bacterial functions should be required to clarify the role of the gut microbiome in CRC development.

Although this study was the first to compare the gut microbiome between two types of CRC precursor, TA and SSA/P, it has several limitations. The small sample size is the first limitation. Although some significant differences in the gut microbiome were identified at the phylum level, statistical power could not be achieved at a deeper level because of the small sample size. Further large-scaled studies are needed for identifying detailed differences of gut microbiome among disease status. Second, all CRCs in our study were located in the distal colon. Unfortunately, patients with proximal colon cancer could not be recruited during the study period therefore we performed a subgroup analysis for lesions located in the distal colon. Subgroup analysis revealed that the relative abundance of bacteria including *Fusobacteria* did not differ according to the tumor location in our study. Finally, although our data showed the comparison of gut microbiome between patients with TA and those with SSA/P, we could not make a definitive conclusion about the role of *Fusobacteria* in serrated pathways. Experimental studies using a SSA/P animal model may provide data supporting our current study.

Despite these limitations, our data provide a better understanding of the role of *Fusobacteria* in the development of CRC. The relative abundance of *Fusobacteria* in CRCs was higher than that in the TAs or SSA/Ps. In addition, the gut microbiome, including relative abundance of *Fusobacteria*, did not differ between the TAs and SSA/Ps. Therefore, *Fusobacteria* may contribute to the serrated pathway as well as the adenoma-carcinoma sequence.

## Methods

### Study population

Patients that were pathologically diagnosed with TA, SSA/P, or CRC were recruited for this study. Written informed consent was obtained from all patients. Patients who were diagnosed with inflammatory bowel disease and human immunodeficiency virus enteropathy were excluded. Additionally, patients who had been treated with antibiotics or antidiarrheal agents within 3 months prior to enrollment were excluded. The study protocol was approved by the Institutional Review Board of Hanyang University Guri Hospital (GURI 2014-10-010). All experiments were performed in accordance with relevant guidelines and regulations.

### Tissue sampling and bacterial DNA extraction

Three pieces of mucosal tissue were obtained from patients with TA, SSA/P, or CRC by endoscopic biopsy. Additionally, four biopsy samples were taken from adjacent normal tissues of each patient.

Extraction of bacterial DNA was performed from mucosal biopsy samples as we previously reported^25^. Briefly, 100 mg of frozen gastric mucosal tissues was suspended in 750 μL of sterile bacterial lysis buffer (200 mmol/L NaCl, 100 mmol/L EDTA [pH 8.0], 20 mmol/L Tris base, 20 mg/mL lysozyme) and incubated at 37 °C for 30 minutes. Then, we added 20 μL of proteinase K and 80 μL of 10% SDS to the mixture and incubated it at 65 °C for 30 minutes. Finally, bead beating was performed for 90 seconds at 5,300 rpm (PRECELLYS 24; Bertin Technologies, Le Bretonneux, France) after adding a 300 mg of 0.1-mm zirconium beads (BioSpec Products, Bartlesville, OK, USA) for finishing homogenization. The homogenized mixture was cooled on ice and then centrifuged at 14,000 rpm for 5 minutes. Bacterial DNA was extracted from the supernatant by phenol/chloroform/iso-amyl alcohol (25:24:1) followed by chloroform/iso-amyl alcohol (24:1), and precipitated by absolute ethanol at −20 °C for 1 hour. The precipitated DNA was suspended in DNase-free H_2_O and cleaned up using a DNA clean-up kit according to the manufacturer’s instructions. Isolated DNA was stored at −80 °C until use in microbial characterization

### Amplification of 16S rRNA gene and sequencing

Extracted gDNA was amplified using primers targeting the V1 to V3 regions of the 16S rRNA gene. For bacterial gDNA amplification, barcoded primers of 27F 5′-CCATCTCATCCCTGCGTGTCTCCGAC**TCAG**-MID-*GAGTTTGATCMTGGCTCAG*-3′, which was consisted of the GS FLX+ adapter sequence A (underlined sequence), linker nucleotides (bolded sequence), multiplex identifiers sequence (MID), and the universal 16S rRNA-specific sequence (italic sequence). The reverse primer 518R 5′-CCTATCCCCTGTGTGCCTTGGCAGTC**TCAG**-MID-*WTTACCGCGGCTGCTGG*-3′ consisted of the GS FLX+ adapter sequence B (underlined sequence), linker nucleotides (bolded sequence), MID, and the universal 16S rRNA-specific sequence (italic sequence). The amplifications were performed at the following conditions: initial denaturation at 95 °C for 5 minutes, followed by 35 cycles of denaturation at 95 °C for 40 seconds, primer annealing at 57 °C for 40 seconds, and extension at 72 °C for 60 seconds; followed by a final elongation at 72 °C for 60 seconds. 16S rRNA PCR products were quantified, pooled, and purified for the sequencing reaction. Sequencing was performed using a 454 Life Sciences Genome Sequencer FLX+ machine (Roche, Florence, SC, USA) according to the manufacturer’s instructions.

### Analysis of 16S rRNA Sequences

Initially generated partial reads of 16S rRNA were trimmed for quality using the standard SFF software tools from Roche/454. Pre-processing and clustering cluding raw read filtering and trimming, reads picking, and OTU clustering using the CD-HIT-OTU^26^. In addition, bacterial 16S rRNA sequence data of the gastric mucosal microbiome were processed through the QIIME pipeline^27^. Reads which showed low quality, contained incorrect primer sequences, or contained more than one ambiguous base were excluded. The remaining reads were classified into CRC, tubular adenoma, and serrated adenoma groups based on their unique nucleotide barcodes. Chimeric sequences were filtered out using the UCHIME algorithm after nearest alignment space termination (NAST) based on the SILVA database^28^,^29^. Taxonomic composition from phylum to species levels and bacterial diversity for each sample were evaluated based on 97% similarity using the QIIME^30^. A UniFrac distance was calculated from representative reads of each OTU using the UniFrac program^31^. Additionally, Unifrac and weighted Unifrac distances were fed into a principal coordinate analysis (PCoA)^31^.

DNA sequences obtained from this metagenomic project have been deposited in the NCBI Short Read Archive under the Accession No. SRA306790.

### Metagenome prediction

Functional composition of the microbiome was predicted using phylogenetic investigation of communities by reconstruction of unobserved states (PICRUSt)^12^. PICRUSt uses evolutionary modeling to predict metagenomes from 16S data compared with a reference genome database^12^. Metagenome prediction in this study was conducted using Galaxy, which is an open, web-based platform for computational biomedical research (http://huttenhower.sph.harvard.edu/galaxy)^12^. To evaluate dissimilarity between reference genomes and the metagenome, the nearest sequenced taxon index (NSTI), which is the sum of phylogenetic distances for each organism in the OTU table to its nearest relative with a sequenced reference genome, was calculated^12^. NSTI has a negative correlation with accuracy of PICRUSt. Functions of predicted metagenomes were classified according to the Kyoto Encyclopedia of Genes and Genomes (KEGG).

### Statistical analysis

Baseline patient characteristics and results of 16S rRNA gene pyrosequencing were described as median with interquartile range (IQR) or number with proportion. Fisher’s exact test or Kruskal Wallis test was used for group comparisons. Relative abundance of different bacteria phyla in each group was presented as mean with standard deviation. Assigned OTUs were compared individually between study groups using Mann-Whitney U test with false discovery rate (FDR) control to correct for multiple comparisons^32^. All statistical procedures were conducted using R (version 2.15.3; R Foundation for Statistical Computing, Vienna, Austria).

## Additional Information

**How to cite this article**: Park, C. H. *et al.* Role of *Fusobacteria* in the serrated pathway of colorectal carcinogenesis. *Sci. Rep.* **6**, 25271; doi: 10.1038/srep25271 (2016).

## Supplementary Material

Supplementary Dataset 1

## Figures and Tables

**Table 1:** Baseline characteristics and microbiome reads.

Variable	TA	SSA/P	CRC	*P*-value
n	8	10	8	
Age, year, median (IQR)	68 (55–74)	54 (49–66)	63 (60–76)	0.119
Male, n (%)	6 (75.0)	5 (50.0)	7 (87.5)	0.244
Tumor location, n (%)				0.006
Proximal colon	5 (62.5)	7 (70.0)	0 (0.0)	
Distal colon	3 (37.5)	3 (30.0)	8 (100.0)	
Read count, median (IQR)	5,540 (3,150–6,875)	6,162 (4,483–7,394)	4,969 (1,642–6,132)	0.247
OTU, median (IQR)	45.0 (33.3–71.5)	44.0 (28.0–74.5)	55.0 (33.8–80.3)	0.847
Chao1 estimator, median (IQR)	46.8 (36.9–88.5)	48.6 (30.8–81.0)	65.4 (44.8–88.8)	0.551
Shannon’s diversity index, median (IQR)	3.22 (2.89–3.75)	3.51 (2.55–4.00)	3.56 (2.52–4.21)	0.912
Simpson’s diversity index, median (IQR)	0.82 (0.78–0.86)	0.84 (0.70–0.90)	0.84 (0.69–0.91)	0.872

TA, tubular adenoma; SSA/P, sessile serrated adenoma/polyp; CRC, colorectal cancer; OTU, operational taxonomic unit; IQR, interquartile rnage.

**Table 2:** Relative bacterial abundance of gastric mucosa at the phylum level according to histology.

	TA		SSA/P		CRC		TA vs. SSA/P		TA vs. CRC		SSA/P vs. CRC	
	Mean	SD	Mean	SD	Mean	SD	*P*-value	FDR	*P*-value	FDR	*P*-value	FDR
Acidobacteria	0.10%	0.31%	0.01%	0.04%	0.00%	0.00%	0.747	>0.999	>0.999	0.867	>0.999	>0.999
Armatimonadetes	0.00%	0.00%	0.00%	0.00%	0.00%	0.00%	N/A	N/A	N/A	N/A	N/A	N/A
Candidate_division_TM7	0.02%	0.07%	0.00%	0.00%	0.00%	0.00%	0.409	>0.999	>0.999	0.867	N/A	N/A
Chlorobi	0.00%	0.00%	0.00%	0.00%	0.00%	0.00%	>0.999	>0.999	>0.999	0.867	>0.999	>0.999
Chloroflexi	0.00%	0.00%	1.54%	5.56%	0.00%	0.00%	>0.999	>0.999	>0.999	0.867	>0.999	>0.999
Deinococcus-Thermus	0.00%	0.00%	0.00%	0.00%	0.00%	0.00%	N/A	N/A	N/A	N/A	N/A	N/A
Gemmatimonadetes	0.15%	0.44%	0.31%	1.13%	0.00%	0.00%	>0.999	>0.999	>0.999	0.867	>0.999	>0.999
Lentisphaerae	0.00%	0.00%	0.02%	0.07%	0.00%	0.00%	>0.999	>0.999	>0.999	0.867	>0.999	>0.999
Nitrospirae	0.00%	0.00%	0.08%	0.27%	0.00%	0.00%	>0.999	>0.999	>0.999	0.867	>0.999	>0.999
Synergistetes	0.25%	0.74%	0.07%	0.24%	0.00%	0.00%	0.443	>0.999	0.471	0.867	>0.999	>0.999
TM6	0.00%	0.00%	0.00%	0.00%	0.00%	0.00%	N/A	N/A	N/A	N/A	N/A	N/A
Tenericutes	0.00%	0.00%	0.00%	0.00%	0.00%	0.00%	N/A	N/A	N/A	N/A	N/A	N/A
Verrucomicrobia	0.03%	0.06%	0.43%	1.21%	0.00%	0.01%	0.844	>0.999	0.594	0.867	0.566	>0.999
Spirochaetes	0.00%	0.00%	0.00%	0.00%	0.13%	0.35%	>0.999	>0.999	0.206	0.759	0.133	0.654
Other	1.71%	1.72%	0.66%	0.97%	0.33%	0.57%	0.113	>0.999	0.029	0.141	0.419	>0.999
Cyanobacteria	0.11%	0.22%	0.10%	0.36%	0.42%	1.20%	0.544	>0.999	>0.999	0.867	0.752	>0.999
Actinobacteria	2.34%	2.61%	2.75%	3.65%	1.46%	2.65%	0.830	>0.999	0.417	0.867	0.184	0.654
Bacteroidetes	16.68%	13.07%	12.96%	11.73%	19.42%	13.68%	0.881	>0.999	0.673	0.867	0.204	0.654
Proteobacteria	51.25%	26.71%	52.34%	27.80%	22.02%	16.24%	0.896	>0.999	0.027	0.141	0.013	0.101
Firmicutes	23.10%	18.23%	26.87%	20.50%	22.46%	13.29%	0.794	>0.999	0.815	0.867	0.804	>0.999
Fusobacteria	4.26%	8.24%	1.85%	4.33%	33.75%	23.17%	0.739	>0.999	0.002	0.023	<0.001	0.001

TA, tubular adenoma; SSA/P, sessile serrated adenoma/polyp; CRC, colorectal cancer; FDR, false discovery rate; SD, standard deviation.

**Table 3:** Relative abundance of predicted function for specific KEGG modules (level 1) according to histology.

KEGG modules Level 1	Relative abundance for specific KEGG modules (%)												
	TA		SSA/P		CRC		TA vs. SSA/P			TA vs. CRC		SSA/P vs. CRC	
	Mean	SD	Mean	SD	Mean	SD								*P*-value		FDR	*P*-value	FDR	*P*-value	FDR
Cellular Processes	3.30	0.74	3.01	0.89	2.30	0.34	0.274		0.965	0.010	0.088	0.027	0.208
Environmental Information Processing	14.81	1.39	15.69	1.99	13.77	2.04	0.408		0.965	0.442	0.124	0.068	0.208
Genetic Information Processing	17.65	1.83	17.82	2.31	19.43	1.45	0.965		0.965	0.038	0.088	0.122	0.208
Human Diseases	1.05	0.23	0.90	0.15	0.83	0.07	0.237		0.965	0.021	0.088	0.408	0.341
Metabolism	47.05	1.78	46.78	1.54	48.06	1.19	0.829		0.965	0.195	0.097	0.101	0.208
Organismal Systems	0.66	0.08	0.68	0.12	0.73	0.07	0.696		0.965	0.083	0.088	0.515	0.351
Unclassified	15.48	1.22	15.13	1.26	14.89	0.85	0.573		0.965	0.382	0.124	1.000	0.581

KEGG, Kyoto Encyclopedia of Genes and Genomes; TA, tubular adenoma; SSA/P, sessile serrated adenoma/polyp; CRC, colorectal cancer; SD, standard deviation; FDR, false discovery rate; N/A, not applicable.
